# Retraction Note: Combined inhibition of AKT/mTOR and MDM2 enhances Glioblastoma Multiforme cell apoptosis and differentiation of cancer stem cells

**DOI:** 10.1038/s41598-026-66458-5

**Published:** 2026-08-12

**Authors:** Simona Daniele, Barbara Costa, Elisa Zappelli, Eleonora Da Pozzo, Simona Sestito, Giulia Nesi, Pietro Campiglia, Luciana Marinelli, Ettore Novellino, Simona Rapposelli, Claudia Martini

**Affiliations:** 1 Department of Pharmacy, University of Pisa, Pisa, Italy; 2 Department of Pharmacy, University of Salerno, Fisciano, Italy; 3 Department of Pharmacy, University of Naples Federico II, Naples, Italy

Retraction of: *Scientific Reports* 10.1038/srep09956, published online 21 April 2015

The Editors have retracted this Article.

After publication, concerns were raised about Figure 7a. An editorial investigation determined that there appears to be an overlap between the two middle control group panels, as well as overlap in the ISA27 panels. The Authors provided some data on request, but the Editors were not able to verify it. The Authors explanation for this overlap was also insufficient. The Editors have therefore lost confidence in this Article.

Simona Daniele, Barbara Costa, Eleonora Da Pozzo, Pietro Campiglia, Simona Rapposelli and Claudia Martini agree with this retraction.

Elisa Zappelli, Simona Sestito, Giulia Nesi, Luciana Marinelli and Ettore Novellino did not respond to correspondence from the Editors about this retraction.

